# Editorial: Holobionts cross talks during microbial-mediated stress tolerance in plants

**DOI:** 10.3389/fpls.2024.1377919

**Published:** 2024-02-15

**Authors:** Islam A. Abd El-Daim, Maged M. Saad

**Affiliations:** ^1^ Institute of Biology, Environmental and Rural Sciences (IBERS) Aberystwyth University, Aberystwyth, United Kingdom; ^2^ Department of Microbiology, Soils, Water and Environment Research Institute, Agricultural Research Centre, Giza, Egypt; ^3^ DARWIN21, Center for Desert Agriculture (CDA), Biological and Environmental Science and Engineering Division (BESE), King Abdullah University of Science and Technology (KAUST), Thuwal, Saudi Arabia

**Keywords:** holobionts, plant microbiomes, abiotic, biotic, stress

Plant holobionts, the dynamic ecological systems comprising plants and their associated microbiomes, harbor untapped potential for revolutionizing agriculture (Mesny et al., 2023). Hence, unveiling the intricate cross talk within these systems presents a breakthrough in developing chemical-free alternatives to enhance crop yield, enhance stress tolerance, and combat devastating pathogens (Borrero De Acuña and Bernal, 2021). This editorial explores cutting-edge research that illuminates the biochemical and molecular mechanisms governing holobiont interactions, offering insights into sustainable agricultural practices and environmentally friendly approaches to optimize plant-microbe relationships.

In this Research Topic, invited investigators have shared their insights through five original research articles investigating the world of holobionts and their potential in improving plant resilience against both biotic and abiotic stressors. These contributions offer new findings that enhance our understanding of the biochemical and molecular mechanisms governing cross talk within holobionts.

One key area addressed in the topic explores the beneficial traits of plant growth-promoting bacteria (PGPB). In the dazzling symphony of the plant holobiont, these beneficial bacteria play a significant role in enhancing plant growth, combating diseases, and bolstering stress tolerance. Our Research Topic presents an enchanting selection of original research articles uncovering the hidden depths of holobionts and their potential in bolstering plant resilience against both biotic and abiotic stressors.

For an example, in the study by Kelbessa et al., several bacterial strains, including *Serratia plymuthica*, *S. proteamaculans*, *S. rubidaea*, and *Pseudomonas fluorescens*, exhibited significant antagonistic activity against *Phytophthora colocasiae*, the causal agent of taro leaf blight (TLB). These strains were able to reduce TLB disease development by 88.75% to 99.37% in greenhouse trials. Such findings indicate that these strains could potentially be used as a biocontrol strategy for TLB, offering an alternative to synthetic fungicides and promoting sustainable taro production.

Another research contribution by Mulatu et al. focuses on the biological control of coffee wilt disease (CWD) caused by the fungal pathogen *Fusarium xylarioides*. The researchers isolated and screened different Trichoderma strains as biocontrol agents against CWD. The study identified *T. asperellum* AU131 as a potential bio-fungicide for managing CWD in Arabica coffee production. This research provides practical recommendations for coffee farmers to effectively combat CWD using a Trichoderma-based biocontrol strategy, thereby contributing to the sustainability of coffee production.

Furthermore, Xia et al. investigated the development of biocontrol approaches for citrus diseases, particularly Huanglongbing (HLB) caused by *Candidatus liberibacter*. By characterizing the citrus leaf midribs microbiomes in healthy and infected plants, the researchers identified correlations between HLB symptoms and the presence of specific bacterial genera. Notably, they found that microbes belonging to the genera *Erwinia*, *Pseudomonas*, and *Streptomyces* were involved in the defense process against *C. liberibacter*. These findings pave the way for potential biocontrol agents targeting HLB, offering hope for strategies to combat this devastating citrus disease.

In addition to combating phytopathogens, plant-associated bacteria also contribute to plant resilience against abiotic stresses. Aizaz et al. investigated the ability of halotolerant PGPB strains to enhance salt stress tolerance in wheat. Their study evaluated 81 halophilic bacterial isolates and identified Bacillus subtilis GREB3 as the most promising strain for improving salt stress tolerance in wheat. This research opens avenues for developing sustainable agricultural practices in salt-affected regions.

Plant responses to PGPB are not well characterized as it is thought to be dependent on numerous factors including plant genotype and the associated bacterial strain (O’Callaghan et al., 2022). Hence, a fundamental aspect of understanding plant-PGPB interactions is deciphering the molecular mechanisms underlying these relationships. Hanifah et al. conducted detailed transcriptome profiling to uncover the growth promotion and biocontrol effects of four different PGPB strains in tomato and potato plants. Their results revealed reprogramming of host transcriptional networks in response to PGPB treatments, indicating significant changes in gene expression. Notably, certain transcription factor genes were differentially regulated, highlighting their potential role in plant hormone homeostasis and growth regulation.

Overall, the Research Topic sheds light on the functions of plant holobionts and the potential applications of beneficial microbes in supporting sustainable and eco-friendly agriculture. These studies provide valuable insights into the intricate interactions between plants and their associated microbiomes, aiming to optimize plant health, stress tolerance, and productivity. By furthering our understanding of holobiont cross talk and harnessing the potential of beneficial microbes, we can pave the way for a more sustainable and resilient agricultural future.

## Author contributions

IE-D: Writing – original draft, Writing – review & editing. MS: Writing – original draft, Writing – review & editing.
